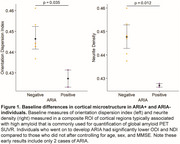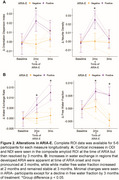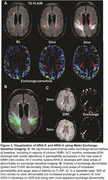# Microstructural and Water Exchange Alterations in ARIA‐E in a Real‐World Cohort Receiving Anti‐Amyloid Therapy

**DOI:** 10.1002/alz70862_110130

**Published:** 2025-12-23

**Authors:** Christopher A Brown, Manuel Taso, Sandhitsu R. Das, Danielle Hing, Emily McGrew, Paul A. Yushkevich, Dawn Mechanic‐Hamilton, Ilya M. Nasrallah, David C Alsop, John A. Detre, David A. Wolk

**Affiliations:** ^1^ University of Pennsylvania, Philadelphia, PA USA; ^2^ Siemens Medical Solutions, Malvern, PA USA; ^3^ Beth Israel Deaconess Medical Center, Boston, MA USA

## Abstract

**Background:**

ARIA‐E represents the greatest risk associated with anti‐amyloid therapy (AAT) and is thought to be due to excessive inflammatory response resulting in FLAIR abnormalities on imaging and occasionally symptomatic manifestations. While FLAIR MRI monitoring for ARIA‐E is now routinely performed, additional MRI contrast mechanisms could provide improved detection and mechanistic insights. We investigated perivascular spaces, microstructural alterations, and parenchymal water permeability in a real‐world cohort receiving AAT using novel MRI techniques.

**Methods:**

Eight participants receiving AAT at the University of Pennsylvania underwent advanced imaging paired with clinical safety MRIs at baseline and during treatment. Advanced imaging sequences included multi‐shell diffusion MRI to assess microstructure, an ultralong TE T2‐weighted sequence to evaluate perivascular spaces structures, and a novel T2‐saturation based water‐exchange sequence. These sequences add <15 minutes to the MRI protocol and can be performed on clinical 3T scanners. We measured microstructure in a composite ROI consisting of cortical regions typically associated with high amyloid deposition as well as in global white matter. Water exchange was calculated specifically in regions that developed ARIA.

**Results:**

2 patients developed ARIA‐E after 2 months of treatment that prompted suspension of treatment: (1) mild symptomatic case that went on to develop mild ARIA‐H at 3 months with resolution of ARIA‐E, (2) moderate case that progressed to severe ARIA‐E at 3 months. At baseline these individuals had lower Neurite Density Index (NDI) and Orientation Dispersion Index (ODI) compared to no ARIA (ARIA‐) patients (Figure 1). At 2 months, they had marked increases in NDI and ODI that resolved by 3 months (Figure 2A). In contrast, there was gradual increase in water exchange and white matter free water fraction (FWF) at 2 and 3 months compared to ARIA‐ (Figure 2B). ARIA‐E and ARIA‐H is also readily visualized on water exchange sensitive maps (Figure 3). Quantification of perivascular volume is ongoing.

**Conclusion:**

Measures of water diffusion and exchange enhance our understanding of ARIA with findings consistent with transient increases in cellularity at ARIA onset and prolonged increases in parenchymal water permeability and edema. Early results suggest microstructure could predict risk for future ARIA. Additional data collection continues.